# Supplementary material for: Silver(I) Complexes Based on Oxadiazole-Functionalized α-Aminophosphonate: Synthesis, Structural Study, and Biological Activities
Source: Molecules. 2022 Nov 22;27(23):8131. doi: 10.3390/molecules27238131 (PMC9738469; doi:10.3390/molecules27238131)
Supplement: Supplementary file 1 [file molecules-27-08131-s001.zip › molecules-2029098-supplementary.pdf]

Silver(I) Complexes Based on Oxadiazole-Functionalized α-Aminophosphonate: Synthesis, Structural Study and Biological Activities

Shaima Hkiri, Kübra Açıkalın Coşkun, Elvan Üstün, Ali Samarat, Yusuf Tutar,
Neslihan Şahin and David Sémeril

Contents

FT-IR spectra of silver complexes **2** and **3** p 2

X-ray structure of complex **2** p 3

X-ray structure of complex **3** p 6

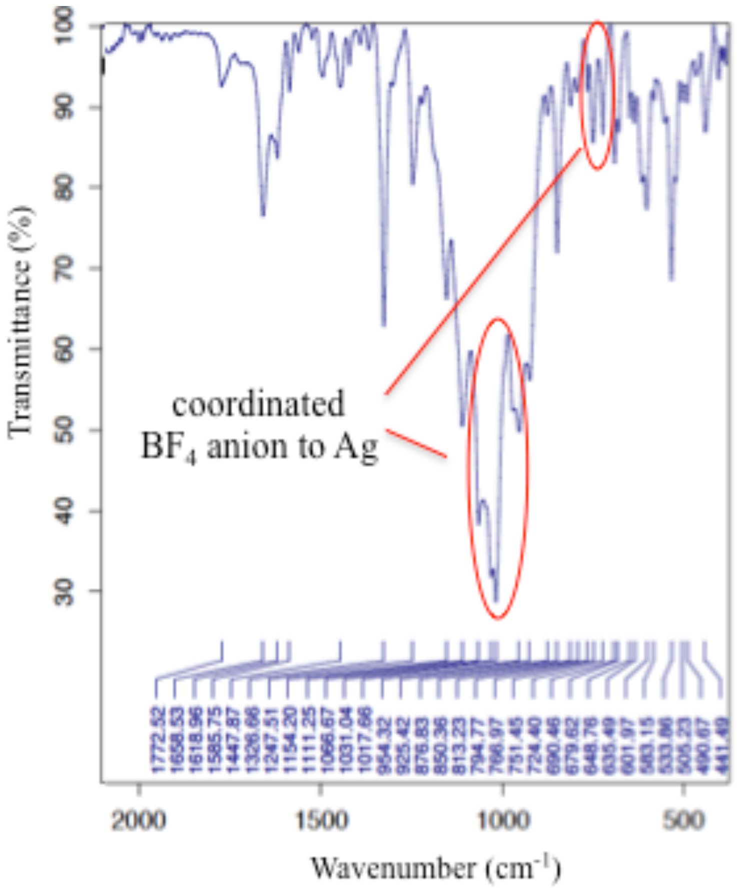

FT-IR spectrum of silver complex **2**.

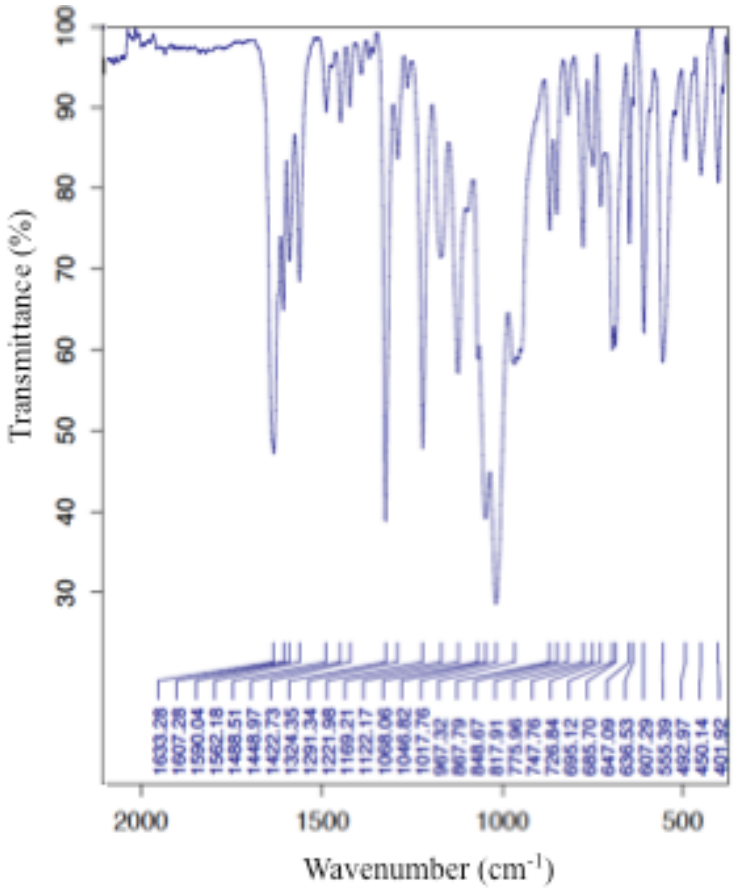

FT-IR spectrum of silver complex **3**.

X-ray structure of complex 2

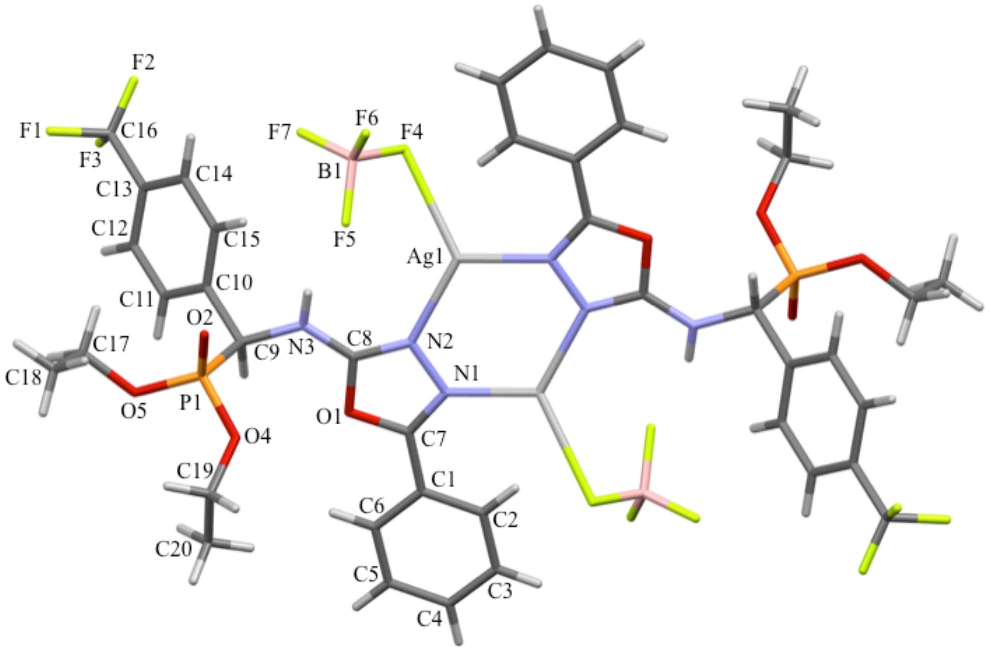

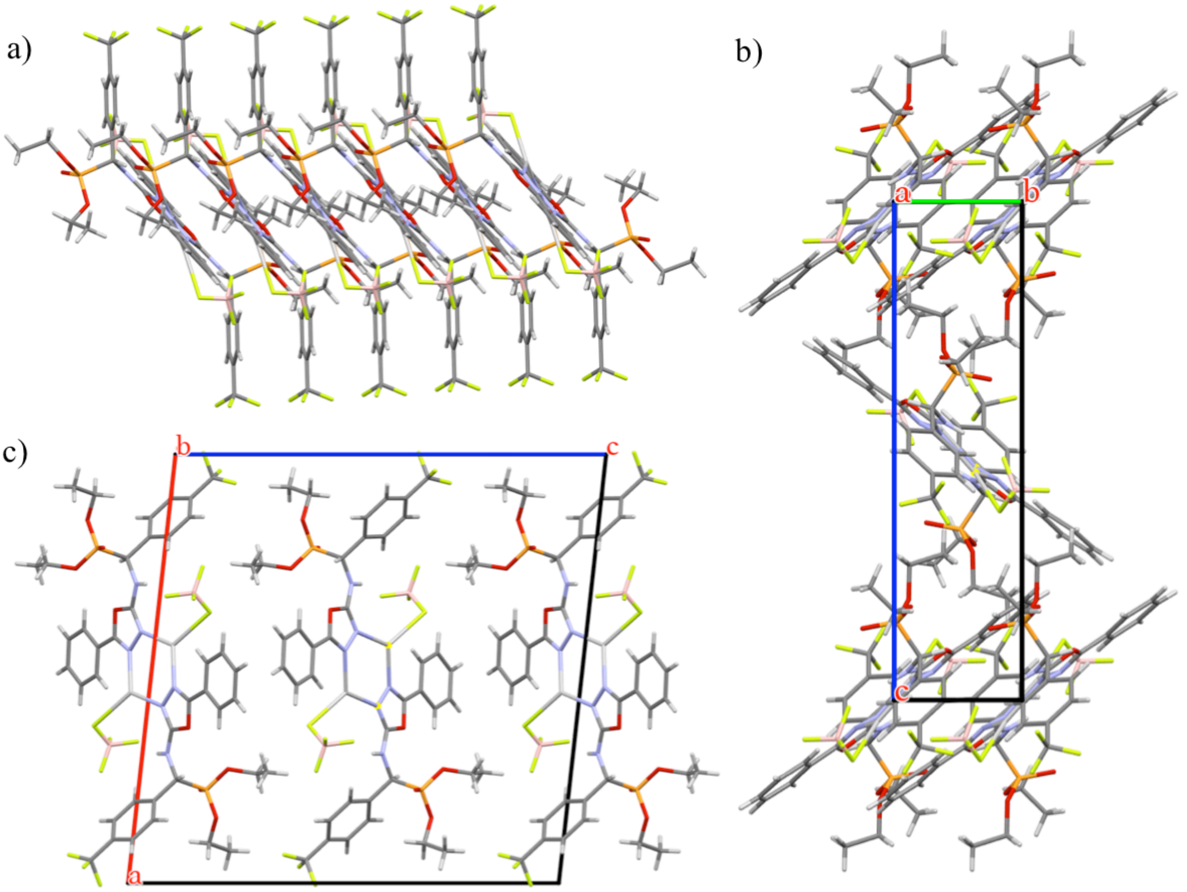

Figure S1. One-dimensional chain of **2** (a); three-dimensional network of **2**: along a axis (b) and along b axis (c) with *a* = 21.2741(8) Å, *b* = 5.3745(2) Å, *c* = 21.0826(9) Å.

Lengths (Å)		
C1 - C6 1.394(4)	C6 - H6 0.9500	C10 - C15 1.387(4)
C1 - C2 1.403(4)	C7 - N1 1.293(4)	C11 - C12 1.392(4)
C1 - C7 1.454(4)	C7 - O1 1.372(3)	C11 - H11 0.9500
C2 - C3 1.387(4)	C8 - N2 1.311(4)	C12 - C13 1.381(5)
C2 - H2 0.9500	C8 - N3 1.325(4)	C12 - H12 0.9500
C3 - C4 1.397(4)	C8 - O1 1.353(3)	C13 - C14 1.381(5)
C3 - H3 0.9500	C9 - N3 1.463(3)	C13 - C16 1.490(4)
C4 - C5 1.392(4)	C9 - C10 1.522(4)	C14 - C15 1.389(4)
C4 - H4 0.9500	C9 - P1 1.827(3)	C14 - H14 0.9500
C5 - C6 1.385(4)	C9 - H9 1.0000	C15 - H15 0.9500
C5 - H5 0.9500	C10 - C11 1.386(4)	C16 - F2 1.270(7)
C16 - F1B 1.357(9)	C19 - O4 1.458(3)	O2 - P1 1.465(2)
C16- F1 1.372(6)	C19 - C20 1.501(5)	O3 - P1 1.572(2)
C16 - F3 1.397(8)	C19 - H19A 0.9900	O4 - P1 1.562(2)
C16 - F2B 1.409(10)	C19 - H19B 0.9900	B1 - F7 1.368(4)
C17 - O3 1.460(4)	C20 - H20A 0.9800	B1 - F6 1.383(4)
C17 - C18 1.505(5)	C20 - H20B 0.9800	B1 - F4 1.402(4)
C17 - H17A 0.9900	C20 - H20C 0.9800	B1 - F5 1.426(4)
C17 - H17B 0.9900	N1 - N2 1.406(3)	F4 - Ag1 2.652(2)
C18 - H18A 0.9800	N1 - Ag1 2.260(2)	Ag1 - N2 2.215(2)
C18 - H18B 0.9800	N2 - Ag1 2.215(2)	
C18 - H18C 0.980	N3 - H3N 0.85(4)	

Angles (°)		
C6 - C1 - C2 119.8(3)	C13 - C12 - H12 120.1	C20 - C19 - H19A 110.0
C6 - C1 - C7 121.0(2)	C11 - C12 - H12 120.1	O4 - C19 - H19B 110.0
C2 - C1 - C7 119.1(2)	C12 - C13 - C14 120.7(3)	C20 - C19 - H19B 110.0
C3 - C2 - C1 120.1(3)	C12 - C13 - C16 120.1(3)	H19A - C19 - H19B 108.4
C3 - C2 - H2 119.9	C14 - C13 - C16 119.3(3)	C19 - C20 - H20A 109.5
C1 - C2 - H2 119.9	C13 - C14 - C15 119.4(3)	C19 - C20 - H20B 109.5
C2 - C3 - C4 119.8(3)	C13 - C14 - H14 120.3	H20A - C20 - H20B 109.5
C2 - C3 - H3 120.1	C15 - C14 - H14 120.3	C19 - C20 - H20C 109.5

C4 - C3 - H3 120.1	C10 - C15 - C14 120.5(3)	H20A - C20 - H20C 109.5
C5 - C4 - C3 120.0(3)	C10 - C15 - H15 119.7	H20B - C20 - H20C 109.5
C5 - C4 - H4 120.0	C14 - C15 - H15 119.7	C7 - N1 - N2 107.4(2)
C3 - C4 - H4 120.0	F3B - C16 - F1B 107.6(5)	C7 - N1 - Ag1 137.61(19)
C6 - C5 - C4 120.5(3)	F2 - C16 - F1 107.8(4)	N2 - N1 - Ag1 110.24(16)
C6 - C5 - H5 119.7	F2 - C16 - F3 108.8(5)	C8 - N2 - N1 105.4(2)
C4 - C5 - H5 119.7	F1 - C16 - F3 99.3(4)	C8 - N2 - Ag1 134.80(19)
C5 - C6 - C1 119.8(3)	F3B - C16 - F2B 102.9(6)	N1 - N2 - Ag1 119.32(16)
C5 - C6 - H6 120.1	F1B - C16 - F2B 102.2(6)	C8 - N3 - C9 124.4(2)
C1 - C6 - H6 120.1	F2 - C16 - C13 117.8(4)	C8 - N3 - H3N 117(2)
N1 - C7 - O1 111.3(2)	F3B - C16 - C13 120.7(5)	C9 - N3 - H3N 119(2)
N1 - C7 - C1 129.7(2)	F1B - C16 - C13 112.6(4)	C8 - O1 - C7 103.6(2)
O1 - C7 - C1 119.0(2)	F1 - C16 - C13 111.6(3)	C17 - O3 - P1 121.0(2)
N2 - C8 - N3 127.5(3)	F3 - C16 - C13 109.9(4)	C19 - O4 - P1 125.18(19)
N2 - C8 - O1 112.3(2)	F2B - C16 - C13 108.8(4)	F7 - B1 - F6 111.8(3)
N3 - C8 - O1 120.1(2)	O3 - C17 - C18 110.9(3)	F7 - B1 - F4 111.5(3)
N3 - C9 - C10 111.2(2)	O3 - C17 - H17A 109.5	F6 - B1 - F4 108.6(3)
N3 - C9 - P1 107.04(19)	C18 - C17 - H17A 109.5	F7 - B1 - F5 110.2(3)
C10 - C9 - P1 111.68(19)	O3 - C17 - H17B 109.5	F6 - B1 - F5 108.1(3)
N3 - C9 - H9 108.9	C18 - C17 - H17B 109.5	F4 - B1 - F5 106.4(3)
C10 - C9 - H9 108.9	H17A - C17 - H17B 108.1	O2 - P1 - O4 116.85(14)
P1 - C9 - H9 108.9	C17 - C18 - H18A 109.5	O2 - P1 - O3 115.03(13)
C11 - C10 - C15 119.5(3)	C17 - C18 - H18B 109.5	O4 - P1 - O3 104.07(12)
C11 - C10 - C9 118.8(3)	H18A - C18 - H18B 109.5	O2 - P1 - C9 112.42(13)
C15 - C10 - C9 121.6(3)	C17 - C18 - H18C 109.5	O4 - P1 - C9 100.16(12)
C10 - C11 - C12 120.1(3)	H18A - C18 - H18C 109.5	O3 - P1 - C9 106.78(12)
C10 - C11 - H11 120.0	H18B - C18 - H18C 109.5	N2 - Ag1 - N1 123.75(8)
C12 - C11 - H11 120.0	O4 - C19 - C20 108.5(3)	N2 - Ag1 - F4 123.02(7)
C13 - C12 - C11 119.7(3)	O4 - C19 - H19A 110.0	N1 - Ag1 - F4 112.85(8)

X-ray structure of complex 3

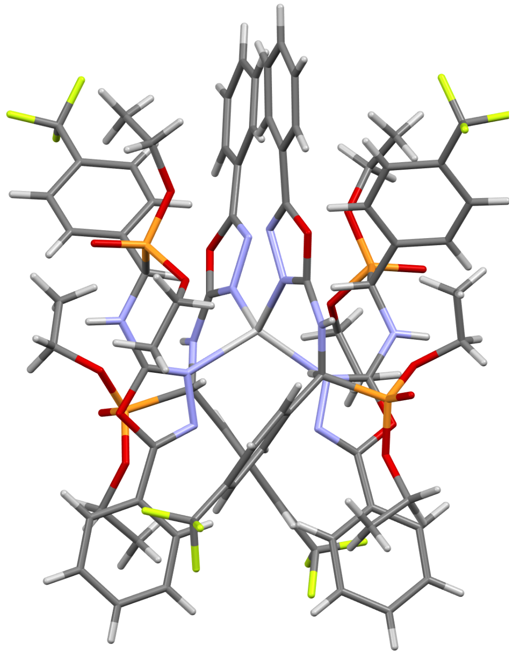

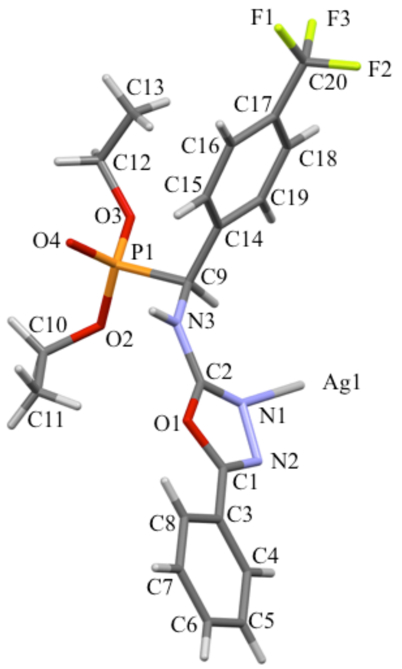

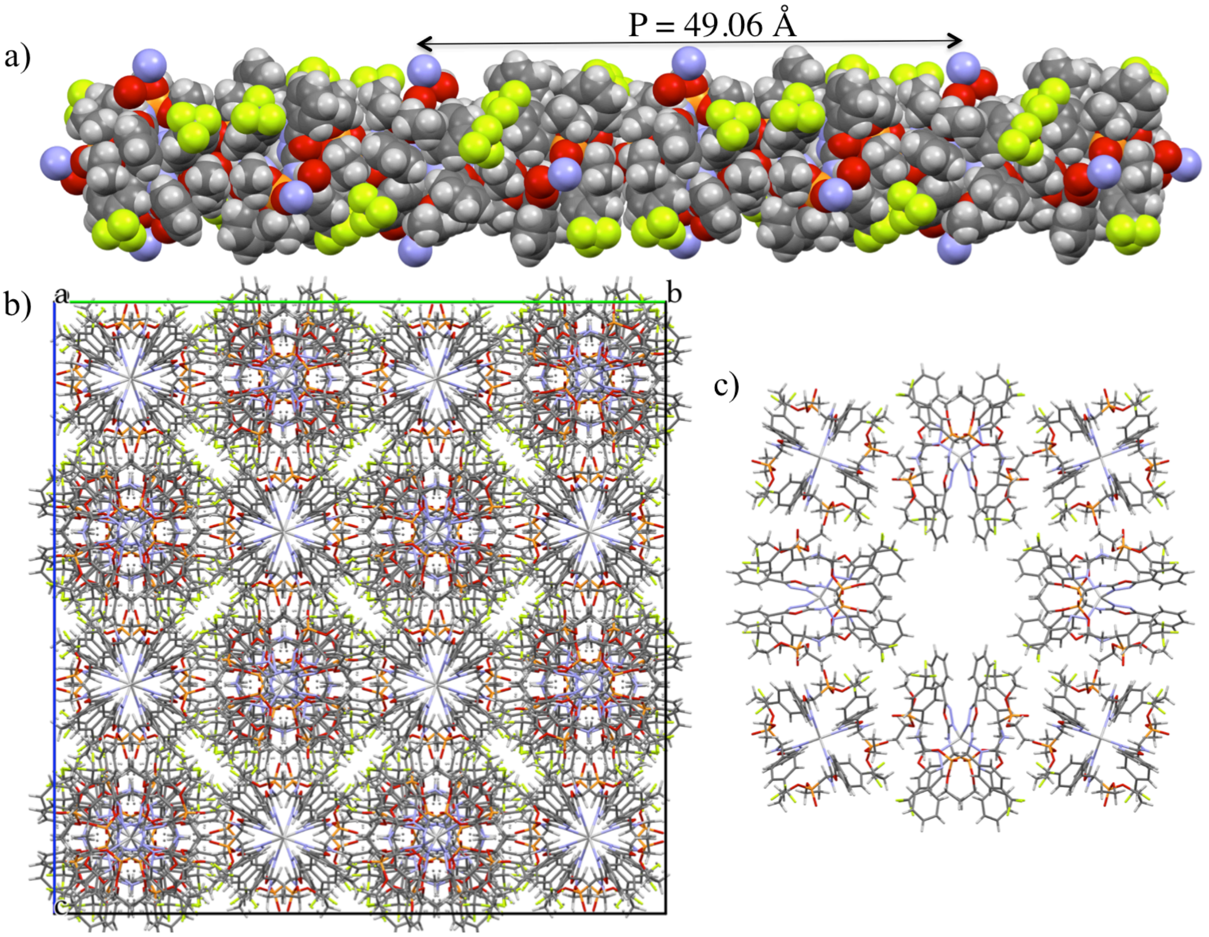

Figure S2. Helical chain observed into **3** complex (a); three-dimensional network of **3** along a axis (b) with a zoom (c); *a* = *b* = *c* = 49.0604(6) Å.

Lengths (Å)		
Ag1 - N1 2.294(5	C7 - C8 1.3900	C13 - H13C 0.9804
Ag1 - N1 2.294(5)	C7 - H7 0.9500	C14 - C15 1.3900
Ag1 - N1 2.294(5)	C8 - H8 0.9500	C14 - C19 1.3900
Ag1 - N1 2.294(5)	N3 - C9 1.460(9)	C15 - C16 1.3900
N1 - C2 1.279(9)	N3 - H3 0.8800	C15 - H15 0.9500
N1 - N2 1.410(7)	C9 - C14 1.488(9)	C16 - C17 1.3900
N2 - C1 1.236(10)	C9 - P1 1.832(8)	C16 - H16 0.9500
C1 - O1 1.353(11)	P1 - O4 1.472(5)	C17 - C20 1.345(13)
C1 - C3 1.478(10)	P1 - O2 1.565(6)	C17 - C18 1.3900
O1 - C2 1.368(8)	P1 - O3 1.570(6)	C18 - C19 1.3900
C2 - N3 1.319(10)	O2 - C10 1.485(11)	C18 - H18 0.9500
C3 - C4 1.3900	O2 - C10A 1.484(14)	C19 - H19 0.9500
C3 - C8 1.3900	C10 - C11 1.502(15)	C20 - F2 1.354(15)
C4 - C5 1.3900	O3 - C12 1.492(13)	C20 - F3A 1.389(17)
C4 - H4 0.9500	C12 - C13 1.457(14)	C20 - F1A 1.404(16)
C5 - C6 1.3900	C12 - H12A 0.9900	C20 - F3 1.415(15)
C5 - H5 0.9500	C12 - H12B 0.9900	C20 - F2A 1.444(16)
C6 - C7 1.3900	C13 - H13A 0.9804	C20 - F1 1.440(15)
C6 - H6 0.9500	C13 - H13B 0.9804	

Angles (°)		
N1 - Ag1 - N1 105.50(12)	N1 - Ag1 - N1 105.50(12)	C2 - N1 - N2 105.6(6)
N1 - Ag1 - N1 117.8(3)	N1 - Ag1 - N1 117.8(3)	C2 - N1 - Ag1 140.2(5)
N1 - Ag1 - N1 105.50(12)	N1 - Ag1 - N1 105.49(12)	N2 - N1 - Ag1 113.9(4)
C1 - N2 - N1 107.6(7)	O2 - P1 - C9 101.1(3)	C12 - C13 - H13B 109.8
N2 - C1 - O1 112.7(7)	O3 - P1 - C9 104.8(3)	H13A - C13 - H13B 109.4
N2 - C1 - C3 125.4(10)	C10 - O2 - P1 119.6(9)	C12 - C13 - H13C 109.1
O1 - C1 - C3 121.8(8)	C10A - O2 - P1 124.3(13)	H13A - C13 - H13C 109.4
C1 - O1 - C2 102.9(6)	O2 - C10 - C11 112.1(11)	H13B - C13 - H13C 109.5
N1 - C2 - N3 131.9(7)	O2 - C10 - H10A 109.2	C15 - C14 - C19 120.0
N1 - C2 - O1 111.2(7)	C11 - C10 - H10A 108.4	C15 - C14 - C9 121.3(4)
N3 - C2 - O1 116.9(6)	O2 - C10 - H10B 109.7	C19 - C14 - C9 118.6(4)

C4 - C3 - C8 120.0	C11 - C10 - H10B 109.5	C14 - C15 - C16 120.0
C4 - C3 - C1 120.6(8)	H10A - C10 - H10B 107.9	C14 - C15 - H15 120.0
C8 - C3 - C1 119.4(8)	C10 - C11 - H11A 109.9	C16 - C15 - H15 120.0
C5 - C4 - C3 120.0	C10 - C11 - H11B 110.0	C15 - C16 - C17 120.0
C5 - C4 - H4 120.0	H11A - C11 - H11B 109.4	C15 - C16 - H16 120.0
C3 - C4 - H4 120.0	C10 - C11 - H11C 108.8	C17 - C16 - H16 120.0
C4 - C5 - C6 120.0	H11A - C11 - H11C 109.4	C20 - C17 - C18 119.3(7)
C4 - C5 - H5 120.0	H11B - C11 - H11C 109.3	C20 - C17 - C16 120.7(7)
C6 - C5 - H5 120.0	O2 - C10A - C11A 112.5(14)	C18 - C17 - C16 120.0
C7 - C6 - C5 120.0	O2 - C10A - H10C 108.4	C17 - C18 - C19 120.0
C7 - C6 - H6 120.0	C11A - C10A - H10C 107.9	C17 - C18 - H18 120.0
C5 - C6 - H6 120.0	O2 - C10A - H10D 109.6	C19 - C18 - H18 120.0
C8 - C7 - C6 120.0	C11A - C10A - H10D 110.5	C18 - C19 - C14 120.0
C8 - C7 - H7 120.0	H10C - C10A - H10D 107.8	C18 - C19 - H19 120.0
C6 - C7 - H7 120.0	C10A - C11A - H11D 112.9	C14 - C19 - H19 120.0
C7 - C8 - C3 120.0	C10A - C11A - H11E 110.7	F2 - C20 - C17 128.6(14)
C7 - C8 - H8 120.0	H11D - C11A - H11E 107.8	C17 - C20 - F3A 140(2)
C3 - C8 - H8 120.0	C10A - C11A - H11F 110.4	C17 - C20 - F1A 116.1(17)
C2 - N3 - C9 122.5(6)	H11D - C11A - H11F 107.1	F3A - C20 - F1A 96.7(13)
C2 - N3 - H3 118.9	H11E - C11A - H11F 107.7	F2 - C20 - F3 95.9(13)
C9 - N3 - H3 118.5	C12 - O3 - P1 125.5(5)	C17 - C20 - F3 121.0(12)
N3 - C9 - C14 113.9(6)	C13 - C12 - O3 109.3(11)	C17 C20 F2A 110.1(14)
N3 - C9 - P1 107.0(5)	C13 - C12 - H12A 109.9	F3A - C20 - F2A 91.3(12)
C14 - C9 - P1 109.6(5)	O3 - C12 - H12A 110.2	F1A - C20 - F2A 90.5(12)
O4 - P1 - O2 116.3(4)	C13 - C12 - H12B 109.4	F2 - C20 - F1 94.5(13)
O4 - P1 - O3 113.3(3)	O3 - C12 - H12B 109.9	C17 - C20 - F1 116.3(12)
O2 - P1 - O3 106.2(3)	H12A - C12 - H12B 108.2	F3 - C20 - F1 92.0(11)
O4 - P1 - C9 113.9(3)	C12 - C13 - H13A 109.7	
